# Management of a Complicated Crown-Root Fracture by Surgical Extrusion: A Two-Year Follow-Up

**DOI:** 10.7759/cureus.114833

**Published:** 2026-08-20

**Authors:** Subananthini Thangaraju, Vinaykumar G, Sibi Swamy, Vinoo Subramaniam Ramachandran

**Affiliations:** 1 Conservative Dentistry and Endodontics, RVS Dental College and Hospital, Coimbatore, IND

**Keywords:** anterior esthetic rehabilitation, case report, complicated crown root fracture, endodontic management, surgical extrusion, traumatic dental injury (tdi)

## Abstract

Complicated crown-root fractures (CRFs) in the anterior region demonstrate significant restorative and esthetic challenges, particularly when the fracture extends subgingivally. Surgical extrusion is a conservative treatment option that repositions the tooth coronally, facilitating restoration while preserving periodontal health. This case report describes the management of a complicated CRF using a minimally traumatic surgical extrusion technique followed by endodontic and restorative rehabilitation. The two-year follow-up demonstrates favorable periodontal healing, tooth stability, and successful restoration of esthetics and function. This case highlights surgical extrusion as a predictable and minimally invasive alternative for preserving compromised anterior teeth in appropriately selected cases.

## Introduction

Traumatic dental injuries (TDIs) are common clinical conditions that can significantly compromise the functional and esthetic integrity of the dentition. The spectrum of TDIs includes crown fracture, crown-root fracture (CRF), root fracture, and luxation injuries, with the permanent maxillary anterior teeth being the most frequently affected owing to their anatomical position and susceptibility to direct trauma [1]. These injuries often involve varying degrees of damage to the dental pulp, periodontal ligament, cementum, and alveolar bone. These injuries predispose the affected teeth to complications such as pulp necrosis, inflammatory root resorption, ankylosis, and eventual tooth loss if not managed appropriately [2].

CRFs are uncommon TDIs involving the enamel, dentin, and cementum, with or without pulpal involvement, accounting for approximately 5% of traumatic injuries in permanent teeth [3]. Management becomes particularly challenging when the fracture extends subgingivally, as it compromises the supracrestal tissue attachment and hinders proper restoration while increasing the risk of periodontal complications [4].

Treatment options include gingivectomy, apically positioned flap surgery, orthodontic extrusion, surgical extrusion, and extraction, depending on the extent and location of the fracture [5]. Among these, surgical extrusion is a conservative and predictable approach that repositions the tooth coronally, allowing restoration while preserving esthetics and periodontal health [6]. It also offers the advantage of immediate repositioning of the tooth, allowing rapid stabilization and early access for endodontic treatment [7].

The present case report describes the multidisciplinary management of a complicated CRF in the anterior esthetic region using surgical extrusion followed by endodontic and restorative rehabilitation. Clinical and radiographic outcomes over a two-year follow-up are presented to demonstrate the effectiveness of this treatment approach in preserving tooth function and periodontal health.

## Case presentation

A 19-year-old male patient presented to the Department of Conservative Dentistry and Endodontics with the chief complaint of pain and fractured upper front teeth following trauma sustained one day earlier. The patient reported a history of a fall from a bicycle, resulting in trauma to the lips and teeth. There was no history of loss of consciousness, vomiting, or other signs suggestive of head injury. The patient was medically fit with no significant medical history. A tetanus booster dose was administered within 24 hours of injury at a primary health center. The patient gave a history of dull, throbbing, intermittent pain aggravated on biting.

**Figure 1:**
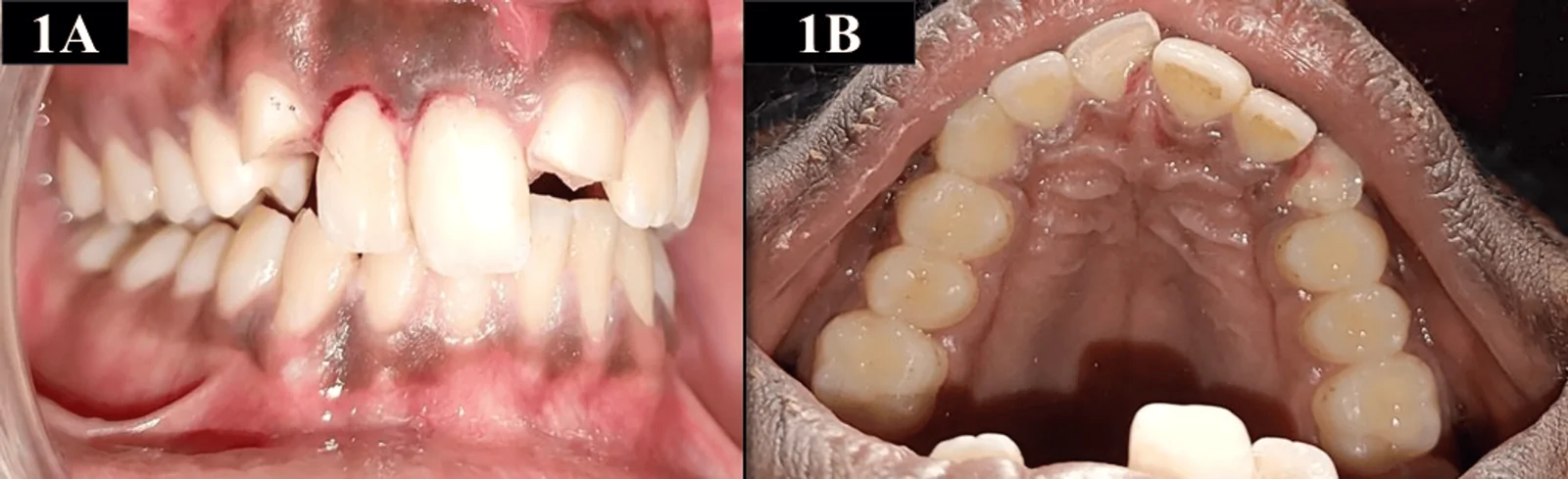
(A) Intraoral frontal view demonstrating a complicated crown-root fracture of tooth 12, an uncomplicated crown fracture of tooth 21, and a complicated crown fracture of tooth 13. (B) Maxillary occlusal view confirming the crown fracture of tooth 13.

Extraoral examination revealed no evidence of facial asymmetry or swelling, and temporomandibular joints demonstrated normal mandibular movements without deviation, clicking, or tenderness. Intraoral soft tissue examination revealed no lacerations, abrasions, or other soft tissue abnormalities. Hard tissue examination demonstrated a complete permanent dentition. Clinical examination revealed a subcrestal CRF of tooth 12, Ellis Class III crown fracture of tooth 13, and Ellis Class II crown fracture of tooth 21 (Figures 1A-1B).

**Figure 2:**
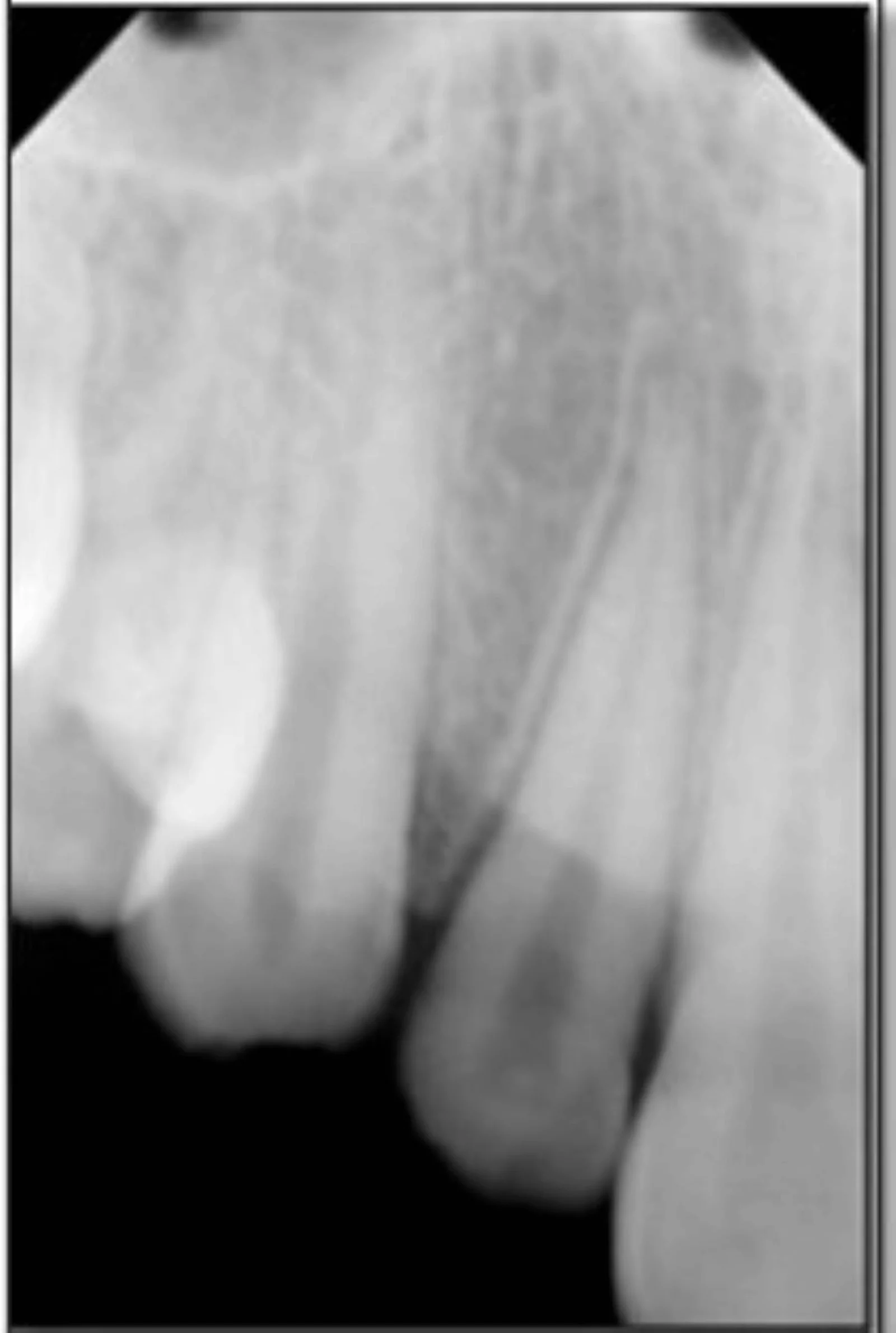
Intraoral periapical radiograph demonstrating widening of the periodontal ligament space and a closed root apex.

Radiographic examination revealed widening of the periodontal ligament space and apical extension of the fracture, which is usually not visible (Figure 2).

The baseline clinical parameters are recorded as follows: pulp sensibility testing was performed using a cold test to determine the status of the pulp, which elicited a positive response in tooth 11, no response in tooth 12, an exaggerated response in tooth 21, and a normal response in tooth 13. The tooth was tender on vertical percussion, with a normal percussion sound in tooth 12.

Based on the clinical and radiographic findings according to the International Association of Dental Traumatology (IADT) guidelines 2020, the final diagnosis was an uncomplicated crown fracture involving tooth 21, a complicated crown fracture involving tooth 13, and a complicated CRF in tooth 12.

Considering the extent of the injury and the subcrestal level of the fracture, a multidisciplinary treatment plan was formulated. The fractured coronal fragment of tooth 12 was removed, followed by surgical extrusion to reposition the tooth coronally, re-establish an adequate ferrule, and preserve the natural tooth. Root canal treatment followed by post-and-core rehabilitation was planned for tooth 13, while composite resin restorations were planned for teeth 12 and 21. Root canal treatment of tooth 12 was not initiated before surgical extrusion because the immediate objective was to atraumatically reposition the subcrestally fractured tooth coronally, thereby exposing the fracture margin for adequate clinical assessment, stabilization, and subsequent restoration. The patient was scheduled for periodic clinical and radiographic follow-up to assess periodontal healing, pulpal status, root resorption, periapical status, and the overall treatment outcome.

**Figure 3:**
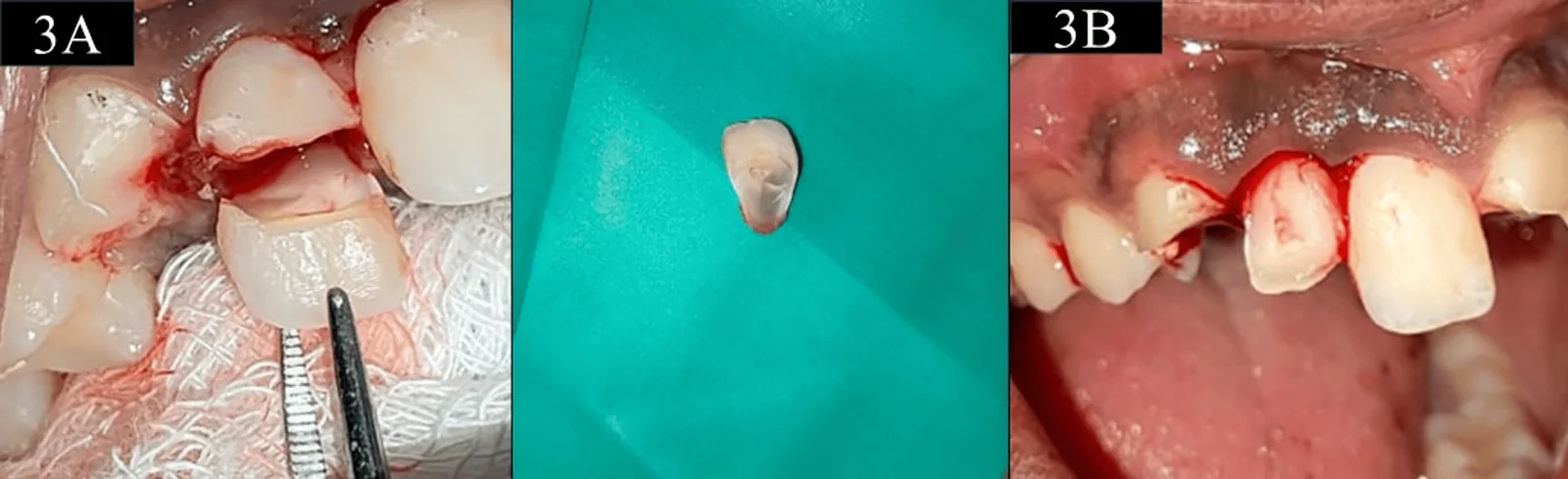
(A) Removal of the fractured crown fragment from tooth 12. (B) Following surgical extrusion, the tooth was rotated 180° to expose the subcrestal fracture margin.

At the first appointment, local anesthesia was achieved with lidocaine 2% with adrenaline 1:100,000 (Lignox 2%, Indoco Remedies Ltd., Mumbai, India), and the fractured crown fragment was removed, followed by surgical extrusion of the tooth without complete removal from the alveolus. The tooth was rotated 180° before repositioning to expose the subcrestal fracture margin circumferentially, facilitating subsequent restoration (Figures 3A-3B) [8].

**Figure 4:**
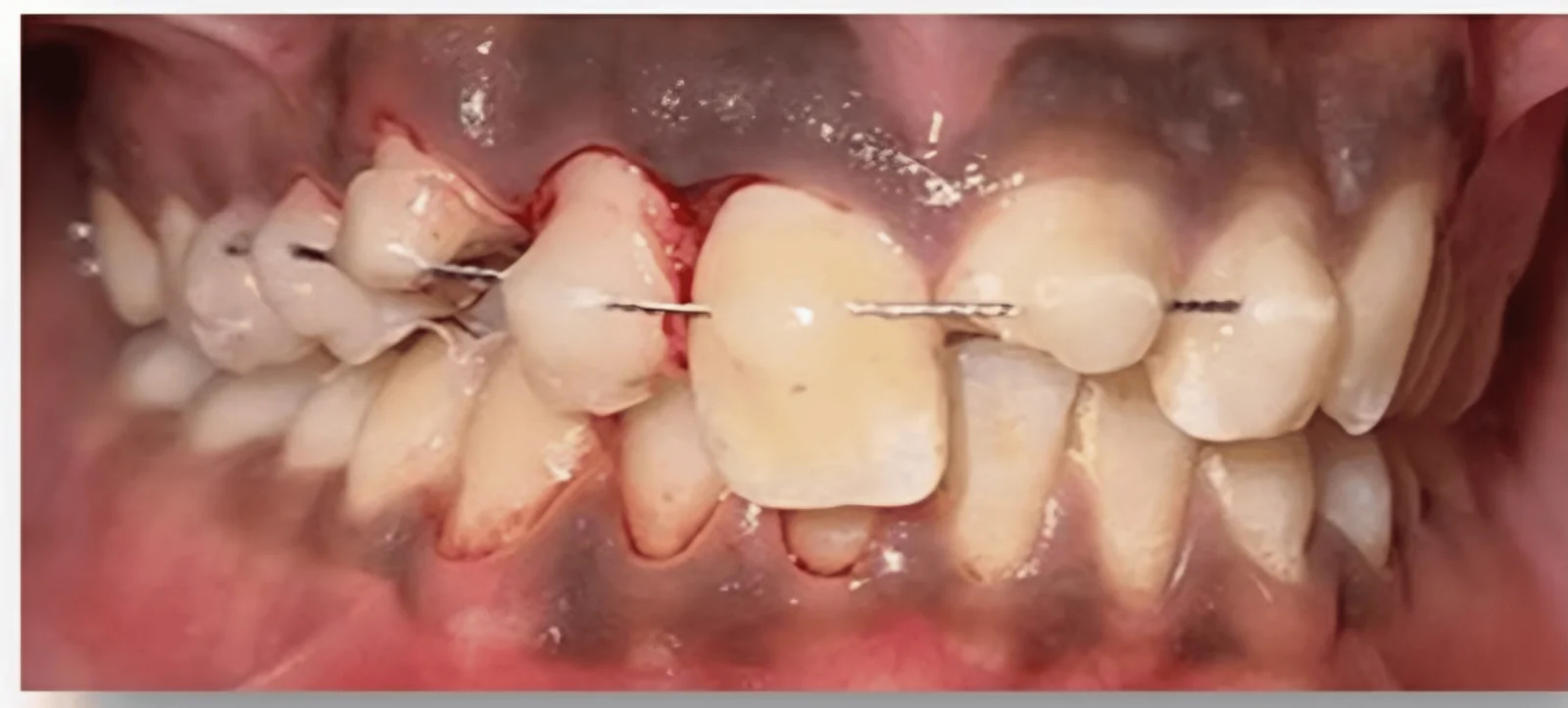
Flexible braided ligature splint bonded with composite resin.

Following surgical extrusion, the tooth was stabilized with a flexible splint fabricated from a 0.010-inch (0.25 mm) braided stainless steel ligature wire to permit physiological tooth mobility and facilitate periodontal healing (Figure 4).

**Figure 5:**
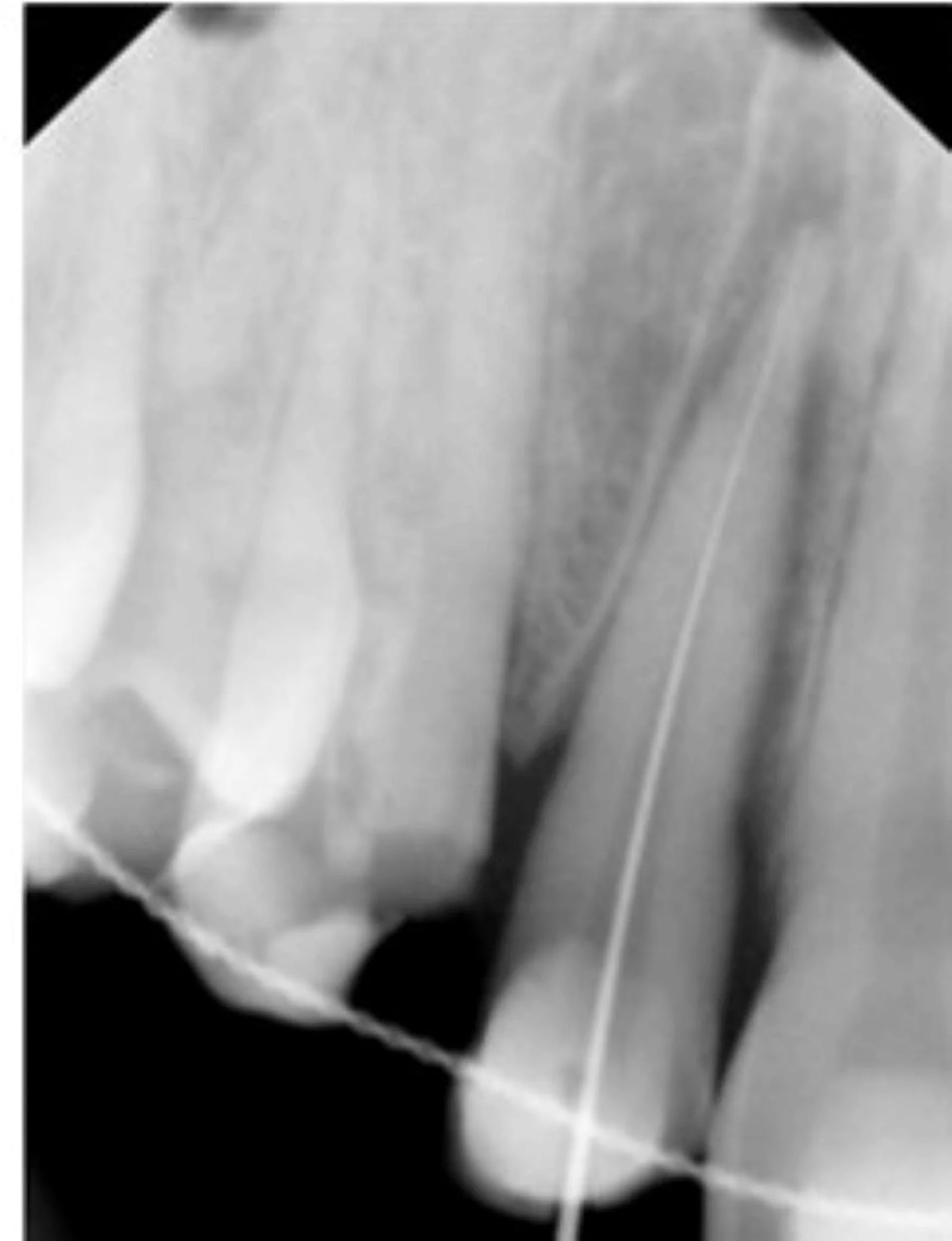
Root canal treatment was initiated during the same visit, and the working length was determined.

Considering the patient's persistent pain and the high risk of pulpal necrosis in a mature permanent tooth, endodontic therapy was initiated during the same visit. Following canal preparation, calcium hydroxide (Ca (OH)_2_) was placed as an intracanal medicament for one week, followed by temporization and subsequently maintained for four weeks to suppress inflammatory root resorption (Figure 5).

**Figure 6:**
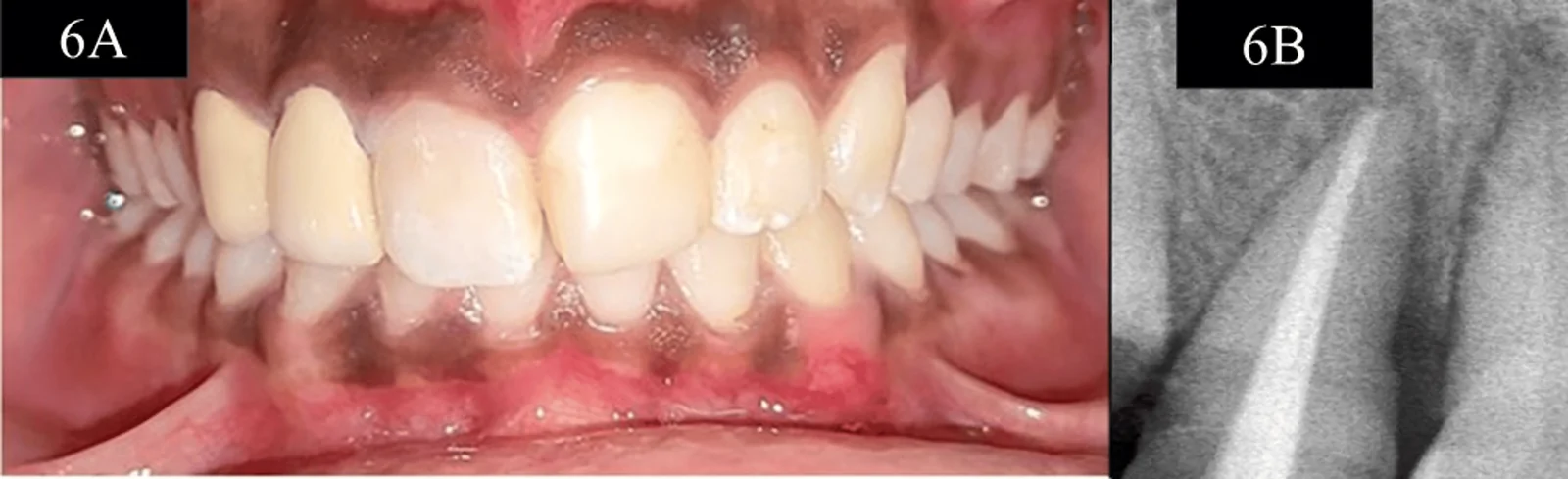
Post-extrusion status and endodontic outcome. (A) Intraoral frontal view after completion of surgical extrusion, showing satisfactory alignment of tooth 12 with an acceptable gingival contour and esthetics. (B) Periapical radiograph confirming root canal obturation, re-establishment of the periodontal ligament space, and absence of pathological root resorption or periapical pathology.

At the recall visit, the patient was asymptomatic, and root canal obturation was completed one month after initiation of endodontic treatment using the cold lateral compaction technique with a bioceramic sealer (CeraSeal, Meta Biomed Co., Ltd., Cheongju, Republic of Korea). Cold lateral compaction was selected because of the mature and well-defined canal anatomy, allowing predictable working length control and controlled apical placement of the gutta-percha. The technique also permits incremental compaction and adaptation of the master and accessory cones, providing a homogeneous and clinically controllable obturation, and controlled spreader pressure was deliberately used to minimize additional mechanical stress on the already compromised root. Following completion of endodontic treatment, tooth 12 was restored with composite resin to provide an adequate coronal seal, followed by definitive rehabilitation with a porcelain-fused-to-metal (PFM) crown at four weeks. Tooth 13 underwent root canal treatment followed by fiber post placement and composite core build-up, and was subsequently restored with a PFM crown at the third week. Tooth 21, which had an uncomplicated crown fracture, was conservatively restored using composite resin. The splinting was removed after four weeks. Throughout the treatment and follow-up period, no clinical or radiographic evidence of inflammatory root resorption, ankylosis, or soft-tissue complications was observed (Figures 6A-6B).

**Figure 7:**
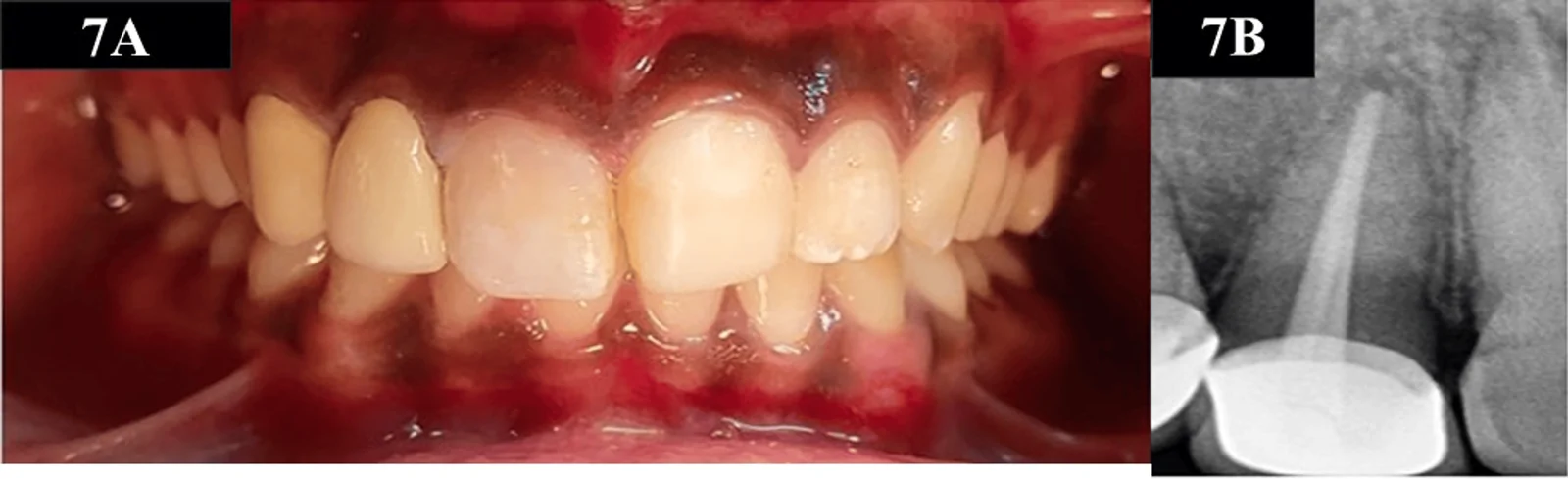
(A) Intraoral clinical photograph showing stable alignment, healthy gingival architecture, and a satisfactory long-term esthetic outcome. (B) Periapical radiograph confirming maintenance of alveolar bone integrity, absence of external root resorption, and favourable periapical status at the two-year follow-up of tooth 12.

At the two-year follow-up, the treated tooth demonstrated favorable clinical and radiographic outcomes. The patient reported no pain, sensitivity, or functional impairment. Clinical examination revealed physiological mobility (Grade 0) and a normal dull percussion sound, with no evidence of ankylosis. Periodontal probing depths were within normal limits (≤3 mm) at all six sites around tooth 12, with no bleeding on probing or gingival recession. The gingival margin remained symmetrical with the contralateral maxillary incisor, and occlusal evaluation demonstrated stable intercuspation without premature contacts. Radiographic examination showed normal periapical status with no evidence of inflammatory root resorption, replacement resorption, or other periapical pathology (Figures 7A-7B).

The timeline of the management of complicated CRF is mentioned in Table 1.

**Table 1:** Chronological timeline of clinical events from the initial trauma presentation to the two-year follow-up. PFM: porcelain-fused-to-metal

Time point	Treatment performed
Day 0: Initial presentation	Tooth 12: Fractured coronal fragment removed, followed by surgical extrusion with 180° rotation of the root; flexible splint placed. Tooth 13: Crown fracture identified and endodontic treatment planned. Tooth 21: Crown fracture identified and composite restoration planned.
One week	Tooth 12: Calcium hydroxide intracanal medicament placed as part of endodontic treatment.
Three weeks	Tooth 13: Root canal treatment completed, followed by fiber post and composite core restoration; PFM crown placed. Tooth 21: Composite resin restoration completed.
Four weeks	Tooth 12: Root canal obturation completed using cold lateral compaction with bioceramic sealer, followed by composite resin restoration and PFM crown placement.
Two-year follow-up	All treated teeth remained clinically satisfactory, with the patient asymptomatic and satisfactory functional and periodontal outcomes.

## Discussion

The current treatment modalities for complicated CRFs include completion of root canal treatment and restoration, orthodontic extrusion, surgical extrusion, root submergence, intentional replantation, and extraction. Conventional crown-lengthening procedures are commonly used to restore compromised teeth; however, they may compromise esthetics in the anterior region by altering the gingival margin and interdental papillae [9]. Surgical extrusion provides a conservative alternative by coronally repositioning the tooth while preserving periodontal architecture and maintaining the required supracrestal tissue attachment for definitive restoration. So, surgical extrusion was preferred in the present case. The tooth was surgically extruded using a minimally traumatic technique without flap elevation or bone removal, followed by endodontic intervention and stabilized with a passive flexible splint for four weeks, consistent with the IADT guidelines [10].

The prognosis is influenced by the timely initiation of root canal treatment, appropriate intracanal medication, and optimal splinting. Splinting should permit physiological tooth mobility and be maintained for the shortest duration necessary to minimize the risk of ankylosis. So, a flexible splint was used and bonded with light-cured composite resin for four weeks to stabilize the surgically extruded tooth while allowing physiological mobility [11].

As teeth with complete root formation have a high incidence of pulp necrosis, early root canal treatment is recommended to reduce the risk of inflammatory root resorption [12]. Hence, in our case, root canal treatment was performed using Ca(OH_2_) as intracanal medicament, because of its well-established antibacterial properties and its ability to prevent infection-related inflammatory root resorption [13]. Consistent with the recommendations of the IADT, Ca(OH_2_) was maintained as an interim intracanal dressing before definitive obturation to promote disinfection and optimize healing, especially in luxation injuries [14].

Long-term follow-up is essential after surgical extrusion, as complications such as ankylosis and replacement root resorption may develop several years after the initial injury. In the present case, the two-year follow-up demonstrated a clinically asymptomatic tooth with normal mobility and no radiographic evidence of periapical pathology, external root resorption, or ankylosis, indicating favourable periodontal and periapical healing and long-term treatment success.

The favourable esthetic and functional outcomes observed in the present case further support surgical extrusion as a biologically sound and predictable treatment option for the management of complicated CRF injuries in permanent teeth.

## Conclusions

Surgical extrusion is a conservative and predictable treatment option for managing complicated subgingival CRFs in the anterior esthetic region. When performed with minimal trauma and followed by appropriate endodontic and restorative rehabilitation, it preserves the natural tooth, maintains periodontal health and esthetics, and restores function. Careful case selection and long-term follow-up are essential for achieving favorable clinical outcomes.
